# Right atrial thrombus formation in a dog after successful electrical cardioversion for atrial fibrillation

**DOI:** 10.1111/jvim.16799

**Published:** 2023-06-29

**Authors:** Marta Karn, Brianna M. Potter, Brian A. Scansen, June A. Boon, Viktor Szatmári

**Affiliations:** ^1^ Deparment of Clinical Sciences, College of Veterinary Medicine & Biomedical Sciences Colorado State University Fort Collins Colorado USA; ^2^ Department of Clinical Sciences, Faculty of Veterinary Medicine Utrecht University Utrecht The Netherlands

**Keywords:** anticoagulation, atrial stunning, echocardiography, hemangiosarcoma, hypercoagulation, neoplasia, thrombolysis

## Abstract

Right atrial masses in dogs are commonly diagnosed as malignant tumors. This report describes a dog with a right atrial mass that appeared after successful electrical cardioversion of atrial fibrillation and resolved with antithrombotic treatment. A 9‐year‐old mastiff was presented for acute vomiting, and occasional cough of several weeks' duration. Ultrasonographic and radiographic examinations of the abdomen and chest identified mechanical ileus, as well as pleural effusion and pulmonary edema, respectively. Echocardiography indicated a dilated cardiomyopathy phenotype. During anesthetic induction for laparotomy, atrial fibrillation developed. Electrical cardioversion successfully restored sinus rhythm. An echocardiogram performed 2 weeks later disclosed a right atrial mass, which had not been apparent before cardioversion. Repeat echocardiography after 2 months of clopidogrel and enoxaparin treatment failed to detect the mass. Intra‐atrial thrombus formation is possible after successful cardioversion of atrial fibrillation and should be considered as a differential diagnosis for echocardiographically detected atrial masses.

## 1 INTRODUCTION

Atrial fibrillation is a common pathologic arrhythmia both in dogs and humans.^1^, ^2^, ^3^, ^4^ Its most disastrous complication in humans is thromboembolic stroke, resulting from left auricular clot formation.^3^, ^4^ In contrast to humans, auricular thrombus formation in dogs with atrial fibrillation is extremely rare.^1^, ^2^, ^5^, ^6^ Although electrical cardioversion is commonly performed in humans to restore sinus rhythm and prevent thromboembolic stroke, paradoxically, cardioversion can promote atrial thrombus formation.^7^, ^8^, ^9^, ^10^, ^11^ Because atrial fibrillation in dogs is rarely associated with thrombus formation, and because sinus rhythm after electrical cardioversion is generally short‐lived, particularly in dogs with concurrent structural heart disease, pharmacologic rate control is the most commonly chosen treatment.^1^, ^2^ Atrial thrombus formation has not been documented in dogs with atrial fibrillation undergoing electrical cardioversion.^5^

Echocardiographic detection of a right atrial mass in a dog is commonly associated with malignant neoplasia, with hemangiosarcoma being the most prevalent tumor type.^12^, ^13^, ^14^ We describe a dog that was diagnosed with a right atrial mass on echocardiography 2 weeks after successful electrical cardioversion because of acute atrial fibrillation. Anticoagulation treatment resulted in complete resolution of the right atrial mass within 2 months.

## 2 CASE HISTORY

A 9‐year‐old male neutered mastiff weighing 56 kg was referred for acute vomiting. The dog had vomited 4 to 5 times after eating, which began the night before presentation. In addition, a single episode of collapse 6 weeks earlier was reported. Finally, the owner mentioned occasional cough, which started 3 weeks earlier. Current medications were PO carprofen (2.2 mg/kg q12h) and PO glucosamine with chondroitin sulfate for chronic arthritis. The dog had a history of 3 previous gastrointestinal foreign body surgeries—an enterotomy, a gastrotomy, and resection of approximately 60 cm of jejunum because of extensive adhesions, 3 years before the current presentation.

Abdominal radiographs completed by the primary care veterinarian showed signs of mechanical ileus as evidenced by radiodense intestinal foreign material with extensive intestinal gas formation. The dog was referred to a specialist center where physical examination disclosed muffled heart sounds as a new finding. A point‐of‐care thoracic ultrasound examination identified a moderate volume of pleural effusion, subjective cardiomegaly, and suspected left ventricular systolic dysfunction. The dog was referred to our institution for evaluation of the heart and the pleural effusion as well as to address the mechanical ileus.

At presentation, the dog was bright, alert, and responsive with a body condition score of 3/9 and moderate muscular atrophy. Rectal temperature was 38.2°C. Continuous panting with mildly increased respiratory effort was noted. Femoral pulse quality was fair, regular, with a frequency of 168/min. Mucous membranes were pink with capillary refill time of <2 seconds. Cardiac auscultation identified a grade 3/6 left apical systolic murmur. Thoracic auscultation identified decreased lung sounds ventrally. Deep abdominal palpation detected a soft abdomen and mild discomfort.

After placement of an IV catheter, an echocardiogram, blood tests, thoracic and abdominal radiographs, thoracocentesis, and abdominocentesis with laboratory fluid analysis were performed.

Blood tests identified mild hypoproteinemia (4.2 g/dL; reference interval [RI], 5.0‐7.0 g/dL), mild hypoalbuminemia (2.2 g/dL; RI, 3.0‐4.3 g/dL), mild hyponatremia (142 mEq/L; RI, 145‐156 mEq/L), mild hyperchloremia (120 mEq/L; RI, 104‐113 mEq/L), and a mildly increased creatine kinase activity (398 IU/L; RI, 50‐275 IU/L). The pH, bicarbonate, potassium, magnesium, calcium, phosphate, glucose, lactate, creatinine, urea, cholesterol, iron, and bilirubin concentrations, and liver enzyme activities, as well as CBC were within the RI.

Abdominal radiographs confirmed mechanical intestinal obstruction caused by radiodense foreign material. Thoracic radiographs showed pleural effusion and signs of pulmonary edema, consistent with congestive heart failure, and no apparent lung metastases.

Echocardiography indicated a dilated cardiomyopathy phenotype with biventricular enlargement. Moderate pleural and no pericardial effusion was noted. Moderate left ventricular eccentric hypertrophy and moderate systolic dysfunction was noted with a normalized left ventricular diastolic internal dimension of 1.93 (normal, <1.85) and a systolic dimension of 1.15 (normal, <0.93) with a fractional shortening of 19% (normal, 30%‐46%). The left atrium was moderately dilated with a left atrial‐to‐aortic ratio of 1.95 (normal, <1.6; measured according to the “Swedish method”). There was a severe, centrally directed jet of mitral valve insufficiency, suspected to be caused by annular dilatation secondary to eccentric left ventricular hypertrophy. The mitral valve leaflets were mildly thickened and mitral inflow was associated with a restrictive filling pattern. The right atrium and ventricle were subjectively mildly dilated. Mild tricuspid valve insufficiency was present. The great vessels showed no abnormalities. No overt cardiac masses were visualized and trace peritoneal effusion was observed. The hepatic veins were moderately dilated, and the caudal vena cava showed decreased respiratory variation in diameter. Simultaneous ECG showed sinus rhythm with a single ventricular premature complex.

Under ultrasound guidance, thoracocentesis and abdominocentesis were performed and fluid samples were submitted for laboratory examination. Both samples were compatible with modified transudate without apparent neoplastic cells, infectious agents, or signs of inflammation. Carprofen was discontinued, and furosemide (2 mg/kg IV q8h), pimobendan (0.27 mg/kg PO q12h), and maropitant (1 mg/kg IV q24h) were started.

An emergency laparotomy was performed to treat the intestinal obstruction. Before anesthetic induction, methadone (0.2 mg/kg IV), fentanyl (2.5 μg/kg IV), midazolam (0.3 mg/kg IV), and lidocaine (2 mg/kg IV) were administered for sedation. Anesthesia was induced with fentanyl (4 μg/kg IV), midazolam (0.12 mg/kg IV), and etomidate (0.1 mg/kg IV). After endotracheal intubation, anesthesia was maintained with inhaled isoflurane and IV fentanyl (0.1 μg/kg/min continuous rate infusion). During anesthesia, dopamine (5 μg/kg/min), lidocaine (50 μg/kg/min), and dobutamine (2.5 μg/kg/min) were administered. At anesthetic induction, sinus rhythm spontaneously converted into atrial fibrillation with a ventricular response rate of 60 beats per minute. At the same time, arterial blood pressure (measured invasively) decreased from 125/98 (mean, 105) mmHg to 68/35 (mean, 48) mmHg. Atrial fibrillation appeared approximately 35 minutes after the lidocaine bolus. The dog was given magnesium chloride (0.3 mEq/kg, diluted 1:5, given over 15 minutes IV) and potassium chloride (10 mEq at a rate of 0.25 mEq/kg/min IV). The dog was placed in dorsal recumbency, and the thorax shaved on both sides. Electrocardiographic leads from the defibrillator device were attached to the dog's skin, lead II was selected, and synchronization mode was switched on to identify native QRS complexes. A single shock of 75 J (1.3 J/kg) was delivered with immediate restoration of sinus rhythm. Systemic arterial blood pressure increased to 112/72 (mean, 85) mmHg. After administration of prophylactic antibiotics (cefoxitin, 24 mg/kg IV 30 minutes before laparotomy, q90min thereafter) the dog was transferred to the operating room. During a midline laparotomy, an intraluminal foreign body was located at the level of the mid‐jejunum at the site of the previous resection and anastomosis with no evidence of hyperemia or damage to serosa or blood supply. Mild adhesions were present at the site of the previous anastomosis, but there was no evidence of perforation or intestinal necrosis. The obstructive foreign material, which appeared to be 2 rocks, was removed. Recovery from anesthesia was uneventful. The mean systemic blood pressure during the procedure varied between 65 and 81 mmHg. The dog was hospitalized overnight on continuous ECG, fluid treatment, fentanyl (2‐5 μg/kg/h IV), metoclopramide (1 mg/kg/day), gabapentin (6 mg/kg PO q8h), and amantadine (4 mg/kg PO q8h). Surface ECG showed sinus rhythm with occasional ventricular premature complexes throughout the day. Two days after surgery, the dog was discharged from the hospital and a re‐evaluation was planned for 2 weeks later. Oral pimobendan (0.3 mg/kg q12h) and furosemide (1 mg/kg q12h) were prescribed.

**FIGURE 1:**
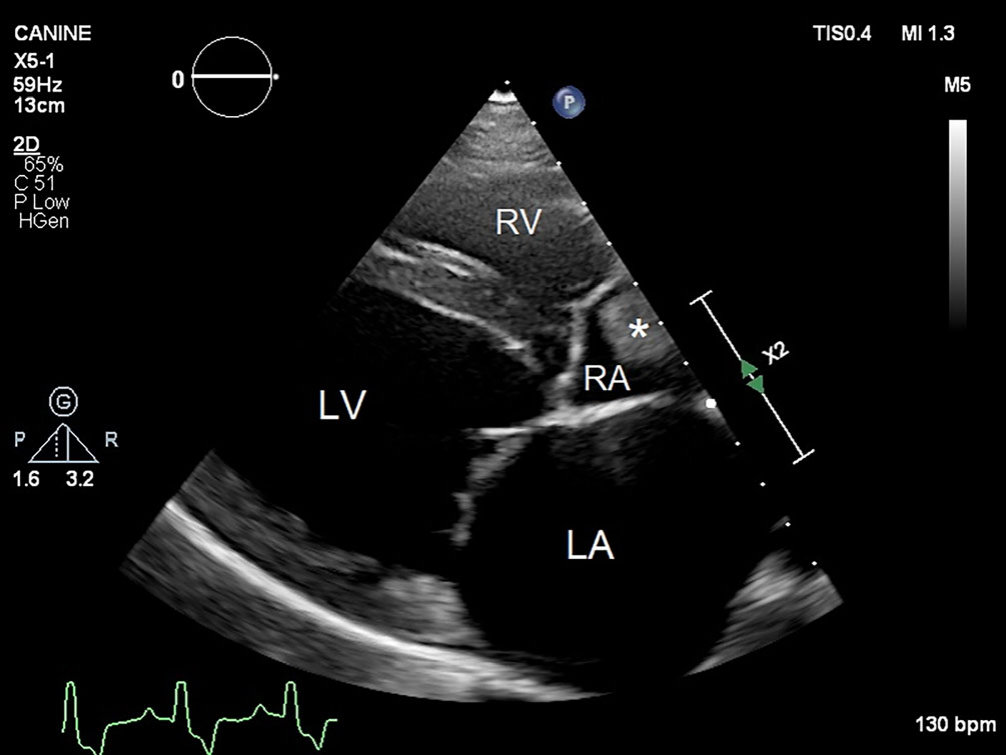
Two‐dimensional echocardiographic image from the right parasternal long axis view shows a moderately dilated left atrium (LA) and left ventricle (LV) and an echogenic rounded soft tissue mass (*) in the mildly dilated right atrium (RA). RV, right ventricle.

**FIGURE 2:**
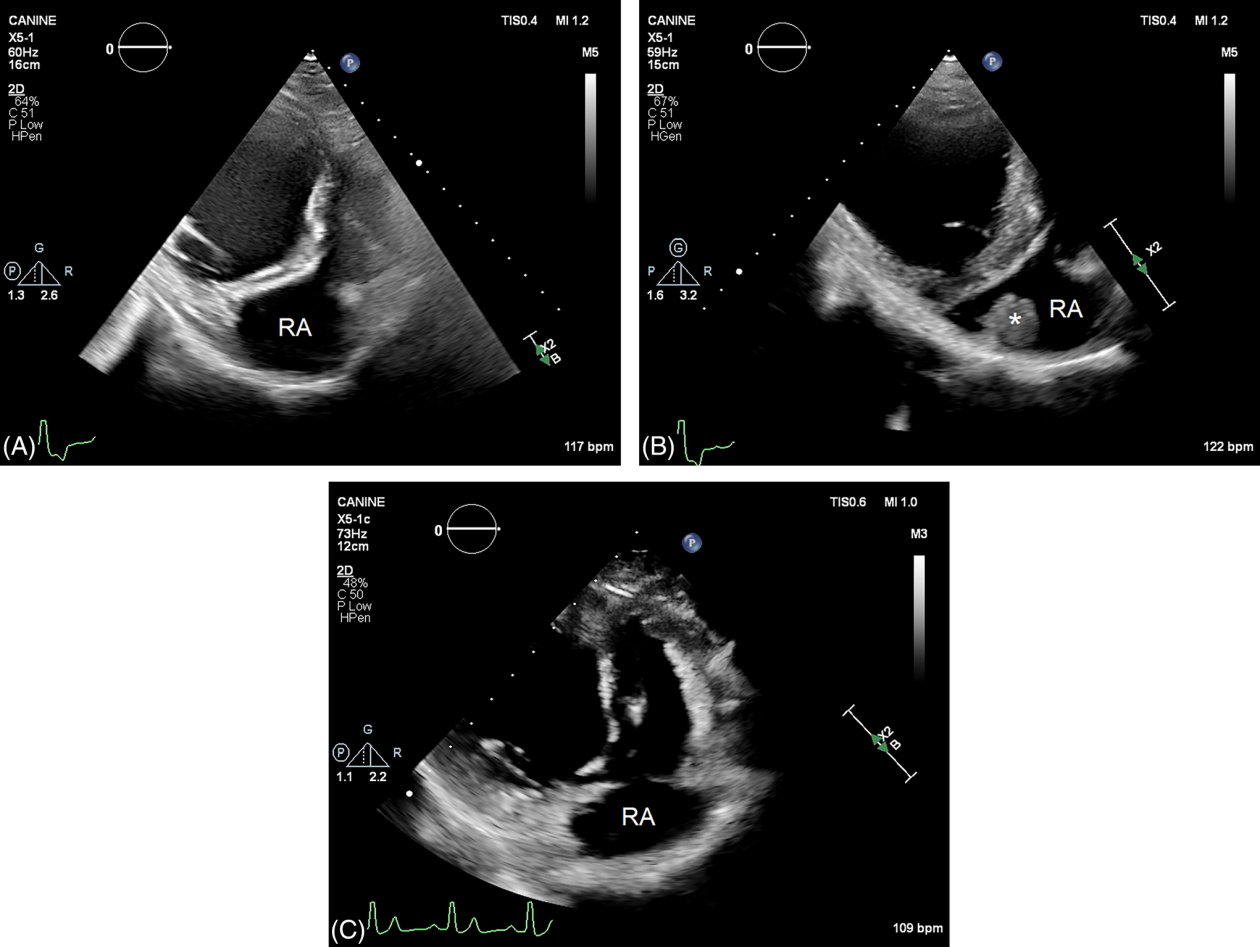
Two‐dimensional echocardiographic image of the right atrium (RA) from the left parasternal cranial view. (A) At presentation: no abnormalities are seen in the right atrium. (B) Two weeks after successful electrical cardioversion, a pedunculated rounded mass with a smooth surface (*) attached to the dorsal right atrial wall is visible in the right atrial chamber. (C) After 2 months of anticoagulation treatment, the right atrial mass is no longer visible.

Echocardiographic examination performed 2 weeks after laparotomy had comparable findings to the preoperative echocardiogram with the exception that no pleural or abdominal effusion was noted. However, an elongated soft tissue mass with a rounded surface and uniform echogenicity was detected in the right atrial lumen attached to the dorsal atrial wall (Figures 1 and 2). The dimensions of this mass were 2 cm × 3.5 cm and the mass did not appear to cause an obstruction to blood flow or distort the function of the tricuspid valve. Simultaneous ECG showed sinus rhythm. Blood tests were performed to help differentiate between a neoplastic process and an intracardiac thrombus. D‐dimer (0.15 μg/mL; RI, 0.03‐0.4 μg/mL) and antithrombin III (142%; RI, 104%‐162%) concentrations were normal, and fibrin degradation products (<5 μg/mL; RI, 0‐4 μg/mL) were negative. High sensitivity cardiac troponin‐I concentration was 0.148 ng/mL (RI, <0.25 ng/mL). Oral clopidogrel (3 mg/kg q24h), enalapril (0.5 mg/kg q12h), and spironolactone (2 mg/kg q24h) were prescribed, and pimobendan and furosemide were continued.

Echocardiography 1 week after starting clopidogrel showed a reduction in the size of the mass (1.8 cm × 2 cm). Enoxaparin was prescribed to be administered as injections by the owner at home (60 mg/0.6 mL syringes, 1 mg/kg q12h SC, 1 syringe per injection). Recheck echocardiography 1 week later showed further decrease in the size of the mass (1.3 cm × 1.7 cm). Echocardiography 2 months after initial detection of the right atrial mass indicated resolution of the thrombus (Figure 2). Enoxaparin was administered for another 2 weeks and then discontinued. All PO medications were continued.

## 3 DISCUSSION

We documented right atrial thrombus formation in a dog after successful electrical cardioversion for atrial fibrillation. In humans, atrial thrombus formation is a well‐recognized complication of cardioversion, occurring in cases of successful cardioversion of atrial fibrillation or atrial flutter.^7^, ^8^, ^9^, ^10^, ^11^ The underlying mechanism is described as atrial stunning, a transient form of atrial mechanical dysfunction.^8^, ^9^ Atrial stunning does not develop after unsuccessful attempts of electrical cardioversion or in the absence of atrial fibrillation or flutter.^8^, ^9^ Atrial stunning develops not only after electrical, but also after pharmacologic and spontaneous conversion of atrial fibrillation to sinus rhythm.^8^, ^9^ Electrical current by itself, even in cases of dilated atria, does not result in atrial stunning.^8^, ^9^ Atrial stunning is maximal immediately after cardioversion and can last for minutes to weeks, but in most cases, it resolves within 3 days.^8^ The incidence of atrial stunning in humans after conversion of atrial fibrillation to sinus rhythm is estimated between 38% and 80%.^8^ Atrial stunning also has been documented in dogs in an experimental study using transesophageal Doppler echocardiography.^7^ The chronicity of atrial fibrillation, atrial size, and underlying structural heart disease are suspected to determine the severity and duration of atrial stunning.^8^, ^9^ The underlying mechanism of atrial stunning is thought to be tachycardia‐induced atrial myopathy.^9^ During atrial fibrillation, the frequent atrial depolarizations result in excessive intracellular calcium accumulation, which in turn leads to desensitization or down‐regulation of the sarcomeric calcium receptors.^9^ Recurrence of sinus rhythm causes a relative calcium deficiency resulting in atrial stunning.^9^ Because of the known risk of development of an atrial thrombus in people who undergo cardioversion for atrial fibrillation or flutter, prophylactic anticoagulation treatment is routinely recommended.^8^, ^9^, ^10^, ^11^ Why atrial thrombus formation in dogs with atrial fibrillation is much less frequent compared to people is unknown.^15^ A study of dogs with atrial fibrillation found no differences in various blood test results before and after electrical cardioversion, but coagulation parameters were not determined.^16^ A possible explanation for the absence of thrombus formation after electrical cardioversion in dogs is that electrical cardioversion is a much less frequently performed procedure in dogs compared to humans and, even when it is performed, postcardioversion echocardiography is not routinely performed. Furthermore, most dogs develop atrial fibrillation secondary to structural heart disease, with severe mitral valve insufficiency being the predominant structural lesion, which may limit blood stasis, lessening thrombotic risk. Thromboembolism arising from the left atrium could lead to cerebral, myocardial, intestinal, or renal infarcts, or ischemic myopathy.^8^, ^9^, ^10^, ^11^ Thromboembolism arising from the right atrium could lead to pulmonary thromboembolism.^8^

Right atrial thrombus in a dog with atrial fibrillation has not been reported previously. In humans with atrial fibrillation, right atrial thrombus formation is very rare and, if it occurs, is typically localized in the atrial appendage.^8^, ^17^, ^18^ A possible explanation for why the thrombus formed in the right atrium instead of the left atrium in our case could be the presence of severe mitral valve regurgitation, which did not allow for stasis of left atrial blood. The dog had no known predisposing diseases for a hypercoagulable state. The intestinal foreign body did not cause peritonitis, as evidenced by cytologic examination of the abdominal effusion. However, surgery, even in the absence of a septic component, can lead to postoperative hypercoagulability.^19^, ^20^ The underlying mechanism is an acute phase response, which can cause increased concentrations of acute phase proteins, including fibrinogen and proinflammatory cytokines.^19^, ^20^ Therefore, the laparotomy might have caused or contributed to right atrial clot formation.

Differentiating a malignant tumor from a thrombus by echocardiography alone can be very difficult, if not impossible, especially if the mass is attached to the atrial wall. Ultrasound‐guided fine needle aspiration biopsy to sample the mass for cytologic examination would not only carry a high risk of inducing arrhythmias and intrapericardial hemorrhage, but it would likely not be able to differentiate thrombus from hemangiosarcoma. In our case, serum cardiac troponin I concentration was measured, because increased concentrations of this cardiac biomarker have been reported in dogs with cardiac hemangiosarcoma.^21^ The serum troponin I concentration was low, which made an invasive cardiac tumor less likely. To investigate the likelihood of a thrombus, D‐dimers and fibrin degradation products were measured, both of which were low.^22^ Because an echocardiogram before cardioversion showed no right atrial mass, it seems unlikely that a malignant tumor grew from undetectable to a 3 cm mass within a 2‐week period. The low D‐dimers concentration might be explained by the relatively small size of the thrombus. When treatment options were evaluated, we considered that a right atrial hemangiosarcoma would represent a highly malignant and incurable tumor, whereas a thrombus potentially could be resolved pharmacologically. Therefore, PO and parenteral antithrombotic treatment was started.^23^ In addition, serial recheck echocardiograms were recommended to monitor the size of the mass, which documented reduction of the mass during the time of antithrombotic treatment. No adverse effects of the treatment were noted. The congestive heart failure was clinically under control with cardiac medications.

Because of the acute onset of atrial fibrillation in the present case, there was no time for pretreating the dog with amiodarone.^1^, ^2^, ^5^ To increase the chance for successful cardioversion, magnesium, and potassium infusions were started.^1^, ^2^, ^24^ Although IV administration of lidocaine is described to convert vagally associated atrial fibrillation in dogs,^25^ our dog had received a bolus of lidocaine shortly before atrial fibrillation developed and was receiving lidocaine as a continuous rate infusion.

To establish the incidence of atrial thrombus formation in dogs after electrical cardioversion, screening echocardiography could be considered within 2 days after successful cardioversion of atrial fibrillation or flutter in future cases.^8^, ^9^, ^10^, ^11^

We conclude that a right atrial thrombus can develop in dogs with atrial fibrillation after successful electrical cardioversion. Because a thrombus can be treated successfully, as opposed to a cardiac hemangiosarcoma, it is important to include this condition on the differential diagnosis, although it is rare.

## CONFLICT OF INTEREST DECLARATION

Authors declare no conflict of interest.

## OFF‐LABEL ANTIMICROBIAL DECLARATION

Authors declare no off‐label use of antimicrobials.

## INSTITUTIONAL ANIMAL CARE AND USE COMMITTEE (IACUC) OR OTHER APPROVAL DECLARATION

Authors declare no IACUC or other approval was needed.

## HUMAN ETHICS APPROVAL DECLARATION

Authors declare human ethics approval was not needed for this study.
